# Newspaper coverage of mental illness in the UK, 1992-2008

**DOI:** 10.1186/1471-2458-11-796

**Published:** 2011-10-12

**Authors:** Robert Goulden, Elizabeth Corker, Sara Evans-Lacko, Diana Rose, Graham Thornicroft, Claire Henderson

**Affiliations:** 1 Section of Community Mental Health, Health Service and Population Research Department, PO Box 29, King's College London, Institute of Psychiatry, De Crespigny Park, London SE5 8AF, UK

## Abstract

**Background:**

Recent years have seen a number of attempts to reduce the stigma related to mental illness; the media can play a significant role in perpetuating this stigma. This paper analyses trends in newspaper coverage of mental illness in the UK between 1992-2008 across a range of psychiatric diagnoses.

**Methods:**

A content analysis was performed on a sample of articles (*n *= 1361) about mental illness in a range of UK newspapers in 1992, 2000, and 2008.

**Results:**

There was a significant proportional reduction in negative articles about mental illness between 1992 and 2008, and a significant increase in articles explaining psychiatric disorders. Coverage improved for depression but remained largely negative for schizophrenia.

**Conclusions:**

Newspaper coverage of mental illness became less stigmatising overall in the 1990s and 2000s, but this was not true for all diagnoses.

## Background

The mass media are strongly implicated in the stigmatising views held by the public towards people with mental illness. The precise nature of mental illness stigma is subject to various definitions, though it is generally held to connote the tainting of affected individuals by an interrelated set of negative ideas (stereotypes) and/or behaviours (discrimination) [1-5]. Common stereotypes include that such individuals are dangerous, unpredictable, incapable, incurable, or strange. The extensive literature on media coverage of mental illness points to its frequent use of such ideas [6-9]. While the relationship between the media and public opinion is to some extent bi-directional, there is evidence of a causal pathway running from negative coverage to prejudicial attitudes [10-13]. In the UK, the past 20 years have seen considerable attempts to challenge stigma and misunderstanding, including the 'Defeat Depression' (1992-1997) and 'Changing Minds' (1998-2003) campaigns of the Royal College of Psychiatrists [14], and awards and guidelines to encourage better coverage of mental illness in the media [15-17]. Longitudinal studies of UK public opinion in this period suggest no overall improvement in attitudes towards mental illness between 1994 and 2003 [18], although there was an improvement in attitudes towards depression between 1991 and 1997 [19]. UK and international surveys find more generally that depression and anxiety disorders are less stigmatised than schizophrenia and other psychotic disorders [20-22]. Most of the research on media coverage is cross-sectional; few studies reveal whether reporting has changed in the long term [23,24] and no studies directly compare reporting of different diagnoses. This study is the first to look at changes in UK newspaper coverage at three time points - 1992, 2000, and 2008 - and at the variation in reporting on a range of diagnoses. We test the hypotheses that: (i) overall coverage will improve across the period, and (ii) that this change will be greater for depression than for schizophrenia. In addition to these specific hypotheses, the study explores the following aspects of coverage: the variation across all common psychiatric diagnoses; the variation across different newspaper types; and the relative weight given to biological and psychosocial models of mental illness.

## Methods

### Sample

The Nexis electronic newspaper archive was used to gather articles from a range of national, general audience dailies. The earliest year for which a broad selection of publications was available was 1992, comprising The Guardian (broadsheet, left-leaning), The Independent (broadsheet, left-leaning), The Times (broadsheet, right-leaning), and The Daily Mail (mid-market tabloid, right-leaning). Only The Guardian and The Times were available before this date, while other national newspapers were not available until the late 1990s. Compared to the UK national newspaper market as a whole, this selection was disproportionately broadsheet and left-leaning, so The Independent was replaced with the London Evening Standard (mid-market tabloid, right-leaning). Excluding The Independent, the 3 national newspapers comprised 30% of the UK national newspaper market as of December 2008. The London Evening Standard was the highest circulation regional newspaper.

The HLEAD function in Nexis was used to search the headlines and opening paragraphs of all articles in the selected newspapers in 1992, 2000, and 2008. The search consisted of 36 general and diagnostic terms covering most major mental disorders (* = truncation wildcard):

General terms: mental health, mental illness, mentally ill, mental disorder, mental patient, mental problem, mental hospital, mental institut*, mental asylum, mental home, secure hospital, special hospital, psychiatr*

Diagnoses: depression, depressive, depressed, anxiety, bipolar, panic disorder, panic attack, obsessive compulsive disorder, OCD, post traumatic stress, PTSD, social phobia, agoraphobi*, schizo*, psychosis, psychotic, eating disorder, anorexi*, bulimi*, personality disorder, dissociative disorder, ADHD, attention deficit

Following Wahl's recommendations [6], substance use disorders, neurodevelopmental and neurodegenerative diseases were not included, as these present a different set of concerns regarding public attitudes and understanding of mental illness. The criteria for inclusion were that the article focused on mental illness, those experiencing it, or the services they receive. Therefore, articles retrieved by the search which used a search term peripherally, in a context unrelated to mental health, or to describe non-clinically significant distress (e.g. casual use of 'depressed'), were excluded.

### Coding

A coding frame was developed based principally on two existing analyses: Corrigan *et al*'s detailed overview of US newspaper coverage [25], and Wahl *et al*'s longitudinal study [24]. Both of these studies had a theoretically-based process of developing codes relevant to mental health stigma, and drew on previous studies and input from advocates in the field. Thematic categories derived from these studies were piloted with a small sample of articles including all three time points, and were accepted, rejected, or adjusted in light of considerations of reliability and validity. This process was carried out by one author (RG), with the final codes reviewed by all authors. Additional file 1: Themes shows the coding frame of the two source studies, and those that were included in this study.

In the finalised coding frame, each article was coded for its primary story theme, whether it mentioned any of four stigma-related elements, and all diagnoses discussed. The story themes were grouped into three categories, and addressed the first hypothesis by looking at *what *is reported. The first of these, 'bad news', included stories considered likely to contribute to mental health stigma. Given the broader range of their content, the 'good news' articles were divided into those explaining and exploring mental illness itself, and those focusing on mental health services and advocacy. In addition to story themes, we used 'elements' to address the first hypothesis by looking at discrete ideas within articles, reflecting more on *how *stories are reported.

The first hypothesis was thus operationalized as a significant reduction in the proportion of articles with 'bad news' themes or containing the fourth element, and a significant increase in the proportion of articles with 'good news' themes or containing the first three elements. A change in these areas meeting a statistical significance threshold of 0.05 would be considered an improvement in reporting. The second hypothesis was operationalized in the same way, as applied to articles featuring depression and schizophrenia, but limited to story themes, as elements were not sufficiently frequent to provide the required statistical power. An improvement in reporting on depression meeting a statistical significance threshold of 0.05, but its absence for schizophrenia, would be considered a greater improvement in reporting for depression.

All articles were coded by the primary rater (RG) using a detailed codebook (see additional file 2: Codebook). A second rater (EC), not involved in developing the coding frame, was trained to perform a reliability check on 10% of the articles using the codebook. After independently coding this sample, the second rater reviewed her coding in consultation with the primary rater, and made adjustments to codes if they were found to directly contradict the codebook on a question of manifest content. Kappa values were calculated to measure agreement.

### Data analysis

The articles were coded and analysed using SPSS version 17.0. The frequency and proportion of articles themes are presented by story year, diagnosis, and newspaper. Mantel-Haenszel chi-squared tests were used to assess the significance of a change across 3 times points and thus investigate longitudinal trends of primary story themes, both overall and for sub-groups of articles by diagnosis and newspaper type. Pearson's chi-squared was used to compare differences in the proportion of negative articles between diagnoses and between newspapers at each time point. The alpha threshold for statistical tests related to the hypotheses was 0.05. For tests not related to the hypotheses, a Bonferroni adjustment for multiple testing was applied, resulting in an alpha threshold of 0.002 (0.05/25).

## Results

The search yielded a total of 7481 articles. A random sequence of 50% of the article numbers was generated from http://www.random.org, and inputted into the Nexis download form to create a random 50% sample. 2380 articles were excluded, primarily due to non-relevant uses of the term 'anxiety', and the use of 'depression' in an economic sense, with both 1992 and 2008 seeing major economic downturns. This resulted in a total of 1361 articles for analysis. The number of relevant articles increased by 35% between 1992 and 2000, and a further 40% between 2000 and 2008, nearly doubling between 1992 and 2008 from 321 to 607.

The reliability check on 10% (*n *= 136) of the articles yielded varying levels of agreement, as measured by the kappa statistic. Agreement was good for diagnosis (0.88) and moderate for story theme (0.64), with the latter increasing when reduced to the three theme groups (0.69) or the binary of 'bad news' or not (0.74). For the stigma-related elements, agreement was highest for 'effective treatment and recovery' (0.81), followed by 'quote from an individual with a mental illness' (0.76), 'pejorative language' (0.66), and 'mental illness is common' (0.64).

### Trends in article content

The frequency and proportion of each of the primary story themes are presented for all three time points (Table 1). There was a significant decline in the proportion of articles containing 'bad news' themes across the sample period (Mantel-Haenszel χ^2 ^= 39.61, d.f. = 1, p < 0.001), countered by a roughly equal rise in the proportion of articles containing 'understanding mental illness' themes (Mantel-Haenszel χ^2 ^= 41.10, d.f. = 1, p < 0.001). There was, however, no trend in the proportion of articles discussing 'services and advocacy' themes (Mantel-Haenszel χ^2 ^= 0.02, d.f. = 1, p = 0.89). Given the absolute increase in the number of articles, this reflects a small increase in the number of 'bad news' articles, a doubling of 'services and advocacy' articles, and a more then threefold increase in 'understanding mental illness' articles.

**Table 1:** Primary story themes of articles on mental illness by year with Mantel-Haenszel chi-squared test for trend.

	Year							
	**1992 (*n = *321)**		**2000 (*n = *433)**		**2008 (*n = *607)**		**Trend**	

**Primary story theme**	**%**	***n***	**%**	***n***	**%**	***n***	**χ^2 ^(d.f. = 1)**	***P***

Bad news	59	188	44	190	37	222	39.606	< 0.001
Danger to others	21	66	23	101	14	87	7.950	0.005
Suicide and self-injury	16	50	5	23	8	48	10.692	0.001
Victimization and severe mistreatment	13	41	4	16	2	11	47.580	< 0.001
Strange, inept, or burdensome	10	31	12	50	13	76	1.627	0.202

Good news								
Understanding mental illness	26	85	45	197	50	301	41.096	< 0.001
Explaining: causes, treatments, prevalence, and symptoms	13	42	29	125	30	182	26.833	< 0.001
Biological	4	13	10	44	7	40	0.761	0.383
Psychosocial	6	20	17	73	19	113	22.151	< 0.001
Not specified	3	9	2	8	5	29	3.765	0.052
Individuals and groups affected by mental illness	13	43	17	72	20	119	5.820	0.016
Services and advocacy	15	48	11	46	14	84	.020	0.888
Mental health service inadequacies and improvements	11	34	8	36	10	61	0.004	0.952
Stigma, discrimination, and public education	4	14	2	10	4	23	0.027	0.871

Among articles categorised as 'bad news', 'danger to others' was the most frequent story type. The proportion of articles coded as 'danger to others' rose between 1992 and 2000 from 21% to 23%, declining to 14% by 2008. At the conservative alpha threshold following Bonferroni adjustment, the decline in 'danger to others' articles was not significant across the period (Mantel-Haenszel χ^2 ^= 7.95, d.f. = 1, p = 0.005). The overall decline in articles in the 'bad news' category is accounted for mostly by a reduction in articles about individuals with a mental illness being harmed by themselves (Mantel-Haenszel χ^2 ^= 10.69, d.f. = 1, p = 0.001) or others (Mantel-Haenszel χ^2 ^= 47.58, d.f. = 1, p < 0.001).

With regards to the 'understanding mental illness' category, the biggest increase was in articles discussing psychosocial causes and treatments, rising from 6% of articles in 1992 to 19% in 2008 (Mantel-Haenszel χ^2 ^= 22.15, d.f. = 1, p < 0.001). Accounts of specific individuals and groups affected by illness rose as a proportion of the total, from 13% to 20%, but this was not significant following Bonferroni adjustment (Mantel-Haenszel χ^2 ^= 5.82, d.f. = 1, p = 0.02). Given that such individual accounts were more likely to touch on psychosocial pathways leading to illness, this further minimizes the relative prevalence of articles discussing mental disorders from a biomedical perspective.

There were few notable changes in the four stigma-related elements of reporting (Table 2). The only significant change was the small increase in articles featuring a quote from individuals with a mental illness (Mantel-Haenszel χ^2 ^= 4.638, d.f. = 1, p = 0.031). In each year the most frequently referenced element was that effective treatment was available and some degree of recovery possible, appearing in around one-fifth of articles. The least frequent element was the use of a pejorative slang term, appearing in 5% or less of articles in each year.

**Table 2:** Elements of articles on mental illness by year with Mantel-Haenszel chi-squared test for trend.

	Year							
	**1992 (*n = *321)**		**2000 (*n = *433)**		**2008 (*n = *607)**		**Trend**	

	**%**	***n***	**%**	***n***	**%**	***n***	**χ^2 ^(d.f. = 1)**	***P***

Notes that effective treatment is available and recovery is possible	16	50	21	90	18	112	0.624	0.429
Notes that mental illness is relatively common	6	18	9	41	5	32	0.484	0.486
Features a direct quote from an individual with mental illness	8	25	12	53	13	78	4.638	0.031
Features a pejorative slang term	5	15	4	18	3	16	2.867	0.090

The proportion of 'bad news' articles decreased for both newspaper types, but with a more significant reduction in the broadsheets (Mantel-Haenszel χ^2 ^= 33.85, d.f. = 1, p < 0.001) than the mid-market tabloids (Mantel-Haenszel χ^2 ^= 9.29, d.f. = 1, p = 0.002). In addition, reporting varied between the two newspaper types at each time point (Table 3). The proportion of articles in the mid-market tabloids which were categorised as 'bad news' was higher than in the broadsheets in each year: this was not significant in 1992 (Pearson's χ^2 ^= 2.04, d.f. = 1, p = 0.15), or 2000 (Pearson's χ^2 ^= 4.47, d.f. = 1, p = 0.035), but highly significant in 2008 (Pearson's χ^2 ^= 14.67, d.f. = 1, p < 0.001).

**Table 3:** Articles by newspaper and proportion of which were bad news with Mantel-Haenszel chi-squared test for trend

	Year							
	**1992 (*n = *321)**		**2000 (*n = *433)**		**2008 (*n = *607)**		**Trend**	

	**%**	***n***	**%**	***n***	**%**	***n***	**χ^2 ^(d.f. = 1)**	***P***

Guardian	37	118	29	125	29	175		
Bad news	59	70	40	50	25	43	35.629	< 0.001

Times	27	87	33	143	32	192		
Bad news	51	44	40	57	36	69	4.908	0.027

Daily Mail	22	72	25	108	25	153		
Bad news	65	47	47	51	45	69	6.736	0.009

Evening Standard	14	44	13	57	13	87		
Bad news	61	27	56	32	47	41	2.595	0.107

a. Newspapers given as % of all articles from specified year. Bad news given as % of all articles in specified newspaper in specified year.

### Variation in coverage by diagnosis

There was considerable variation in article themes by diagnosis (Table 4). However, it should first be noted that in each year of coverage, a large proportion of articles (between 24% and 39%) featured no specific diagnosis. Articles featuring general or non-specific references to mental illness were largely negative in 1992 (76%) and 2000 (73%), but not in 2008 (48%).

**Table 4:** Articles featuring specified diagnosis and primary story theme groups with Mantel-Haenszel chi-squared test for trend.^a, b^

	Year							
	**1992 (*n = *321)**		**2000 (*n = *433)**		**2008 (*n = *607)**		**Trend**	

	**%**	***n***	**%**	***n***	**%**	***n***	**χ^2 ^(d.f. = 1)**	***P***

No diagnosis specified	39	124	24	102	31	189		
Bad news	76	94	73	74	48	91	26.653	< 0.001
Understanding mental illness	6	8	14	14	24	45	17.096	< 0.001
Services and advocacy	18	22	14	14	28	53	5.692	0.017

Depression	28	91	34	149	37	226		
Bad news	38	35	32	47	28	63	3.287	0.070
Understanding mental illness	46	42	59	88	63	142	6.643	0.010
Services and advocacy	15	14	9	14	9	21	1.976	0.160

Eating disorders	14	45	13	58	14	82		
Bad news	58	26	16	9	17	14	20.297	< 0.001
Understanding mental illness	42	19	78	45	78	64	14.682	< 0.001
Services and advocacy	0	0	7	4	5	4	-	-

Anxiety disorders	10	32	14	62	11	67		
Bad news	19	6	21	13	18	12	0.38	0.845
Understanding mental illness	63	20	69	43	72	48	0.760	0.383
Services and advocacy	19	6	10	6	10	7	-	-

Schizophrenia	12	38	14	59	9	57		
Bad news	58	22	49	29	68	39	1.535	0.215
Understanding mental illness	16	6	27	16	25	14	0.758	0.384
Services and advocacy	26	10	24	14	7	4	6.443	0.011

Bipolar disorder	4	12	3	11	3	19		
Bad news	50	6	64	7	21	4	-	-
Understanding mental illness	33	4	9	1	53	10	-	-
Services and advocacy	17	2	27	3	26	5	-	-

Personality disorders	2	7	6	26	1	9		
Bad news	86	6	85	22	56	5	-	-
Understanding mental illness	0	0	8	2	33	3	-	-
Services and advocacy	14	1	8	2	11	1	-	-

ADHD	0	0	4	18	4	24		
Bad news	0	0	22	4	8	2	-	-
Understanding mental illness	0	0	72	13	88	21	-	-
Services and advocacy	0	0	6	1	4	1	-	-

Other disorders	1	2	1	5	1	7		
Bad news	50	1	40	2	14	1	-	-
Understanding mental illness	0	0	60	3	71	5	-	-
Services and advocacy	50	1	0	0	14	1	-	-

a. Diagnoses given as % of all articles from specified year; sum of all diagnoses adds up to more than 100% as articles can feature multiple diagnoses. Primary story theme groups given as % of all articles featuring specified diagnosis from specified year.

b. Test for trend could not be performed for theme groups with expected cell values < 5.

The most frequently covered diagnosis in each year was depression (28% of articles in 1992, 34% in 2000, and 37% in 2008). Most of these articles came under 'understanding mental illness', with the proportion increasing across the period (Mantel-Haenszel χ^2 ^= 6.64, d.f. = 1, p = 0.01). However, the decline in 'bad news' articles about depression fell short of significance (Mantel-Haenszel χ^2 ^= 3.29, d.f. = 1, p = 0.07). Schizophrenia was featured much less frequently than depression, and was more likely to be in the context of 'bad news' when it did appear. Excluding articles in which both diagnoses appeared, there was a significant difference in the proportion of negative articles for schizophrenia and depression in 1992 (Pearson's χ^2 ^= 5.89, d.f. = 1; p = 0.02), 2000 (Pearson's χ^2 ^= 7.42, d.f. = 1, p = 0.006), and 2008 (Pearson's χ^2 ^= 39.64, d.f. = 1, p < 0.001). Following Bonferroni adjustment, however, only the difference in 2008 was significant. There was little improvement in the coverage of schizophrenia over time, with its most frequent story type of 'bad news' seeing no reduction as a proportion across the period (Mantel-Haenszel χ^2 ^= 1.54, d.f. = 1, p = 0.22).

## Discussion

There was a large increase in coverage of mental illness between 1992 and 2008, most of which was not categorised as a 'bad news' story most likely to contribute to the stigma associated with mental illness. However, between 1985 and 2006, the average number of news and editorial pages in UK newspapers increased almost threefold [26]; by this measure, the near doubling in articles about mental illness between 1992 and 2008 likely represents a *decrease *in reporting on this issue as a proportion of all newspaper coverage. Nevertheless, it is significant that while 'bad news' stories about mental illness remain as prevalent in absolute terms as they did nearly 20 years ago, there is now a considerably higher proportion of coverage devoted to explaining mental illness and exploring the experiences of individuals affected by it. The presence of this pattern in all four publications suggests that the observed changes represent a general trend across the print media. This trend in story themes lends support to the first hypothesis, although the much smaller changes in the elements of reporting give some qualification to this finding.

The overall positive trend masks considerable variation by diagnosis. The reporting of depression, anxiety, bipolar disorder, and eating disorders, either improved over time or was always largely favourable. In contrast, schizophrenia, personality disorders, and general references to mental illness, appeared mainly in the context of 'bad news', and saw little or no change in their coverage over time. The significant increase in 'understanding mental illness' articles about depression, and the lack of change in reporting on schizophrenia, lend support to the second hypothesis. However, this finding is also qualified as the decline in 'bad news' articles about depression was not significant.

With regards to the particular story types, the lack of significant change in reporting on danger suggests violent crime remains a popular staple of news media, although less frequent than some previous studies have found [6-9]. In addition to reports on individual crimes and court cases, this theme also covered reporting on issues like the government's introduction of the dangerous and severe personality disorder diagnosis in 2000, which largely explains the small spike in danger stories for that year. It is worth noting that nothing in the results here, or in any other studies, have demonstrated that violent crimes committed by individuals with a mental illness are more likely to be reported than such crimes committed by other individuals. What concerns campaigners more is both *how *such incidents are reported, and the fact that for some illnesses, this seems to be the only time they appear in print. With regards the former, it is encouraging that the use of clearly inflammatory language is quite rare, although other research suggests it is more prevalent in the popular tabloids not featured here [23]. As for the latter concern, we have noted how this is a real problem for schizophrenia and personality disorders, which rarely appear in a context not somehow related to violence, tragedy, or misfortune.

The consistent and increasing preference for reporting on the psychosocial over the biomedical aspects of mental illness raises conflicting issues with regards to stigma. On the one hand, this is a positive change given that the biomedical model of mental disorders may be more likely to illicit stigmatising views from the public [27]. On the other hand, the marginalisation of the biomedical model might simply perpetuate the stigma of those seeking and receiving pharmacotherapy as opposed to psychotherapy.

### Comparisons with previous research

As noted, there are very few other longitudinal studies of newspaper coverage of mental illness for the past 20 years. Clement and Foster found that reporting on schizophrenia in UK newspapers changed little between 1996 and 2005 [23], a finding corroborated by our results. In the US Wahl *et al *found small but positive changes in newspaper coverage between 1989 and 1999, again in broad agreement with our results [24].

### Relationship to campaigns and public opinion

The relationship of these changes in coverage to campaigns and public opinion is undoubtedly complex. This was a study of media content, not of media production or audience reception, and therefore we can not make any specific claims in this area. It is certainly encouraging that a period which began with a major campaign to increase understanding of depression has seen a steady increase in media coverage explaining this disorder. However, establishing a causal role for these campaigns in the observed changes would require consideration of several other variables, such as changes in the accuracy and balance of health reporting more generally.

With regards public opinion, the improvement in views on depression [19], as well as the more negative views held about schizophrenia [20-22], mirrors patterns found in the coverage. However, given the general improvement in coverage, it is surprising that public opinion overall has not changed significantly in the same period [18]. This could be due to a number of factors. Firstly, the "negativity bias" suggests that people pay more attention to bad news than good news [28], with negative reporting thus remaining highly salient even if it is declining in proportional terms. Additionally, television portrayals of mental illness may not have changed over this period, and the public has much more exposure to this medium than newspapers [29]. Finally, there might be a lag in the effects of the print media on public opinion, especially on a topic where many views are so long-held [30].

### Limitations of the study

While this study contributes new information, it has several limitations. Firstly, the variation in coverage across newspapers makes it clear that a more comprehensive sample of the national newspaper market would have been desirable. To this end, it is hoped that tabloid newspaper archives become more easily accessible in future. The study is limited to newspapers, neglecting what may be the more influential messages found in television and film, particularly dramatic fictional portrayals.

Secondly, the use of additional time points would have more clearly revealed the course of the trends observed, and provided greater confidence that no individual events skewed the data. However, given the speed of the news cycle and the fact that the samples were taken from across the whole year, for no time point was a particular story found to dominate coverage.

Thirdly, the fact that this study looked only at articles explicitly *about *mental illness means that it did not analyse many uses of mental health language - both diagnostic and slang - in non-literal contexts. The rationale for excluding such uses is that for all articles to be weighted equally, they had to be of a broadly similar type. Nevertheless, it has been a long standing concern that 'schizophrenic' is often misused [31], and it was clear during the coding process that the term 'psychotic' is often used inaccurately and pejoratively.

Fourthly, there are inherent limitations to quantitative media analyses. In the process of converting complex media messages into a limited number of discreet categories, a considerable amount of meaning is inevitably lost. Most of the story themes contain a diversity of messages. For example, a story coded 'suicide' could be sensationalist, it could be sensitive and sympathetic, or it could be a mix of these. Reliably coding such subjective criteria is extremely difficult, as suggested by our moderate kappa values. This is particularly true for a study such as this which aims to give a broad overview of how all diagnoses are covered, thus requiring somewhat generic categories of story theme.

With regards the statistical analyses, as this study examined trends in media coverage retrospectively, a sample size calculation was not performed and this is a limitation. The magnitude of the chi-square statistic values, however, indicate high levels of significance which allow us to be more confident about our findings.

## Conclusions

This is the first study to look at how UK newspaper coverage has changed through most of the 1990s and 2000s, comparing coverage across a range of psychiatric diagnoses. Those who have struggled to improve understanding of mental illness in recent decades can take encouragement from the improved coverage of depression, eating disorders, and bipolar disorder. But there clearly remains a great deal of work to do for personality disorders and schizophrenia. Given that this study did not look at the metaphorical use of mental health language, television and film portrayals, and the more sensationalist tabloids, the overall coverage of these diagnoses may be worse than it appears here. All this suggests that these disorders should receive particular attention in future campaigns to reduce mental health stigma. Given the relative infrequency of coverage of schizophrenia, the problem is more that there is an *absence *of explanatory and health service-related articles about this illness, than there is an abundance of negative articles. Attempts to encourage such 'positive' reporting might, therefore, be the most fruitful avenue for campaigners.

Finally, given the limitations noted, there is clearly scope for more longitudinal studies, to see if similar patterns are found in television and film portrayals of mental illness, as well as more qualitative comparisons of particular story types. Ideally, such research should be linked with audience reception studies, to see how changes in coverage might translate into changes in public opinion.

## Competing interests

The authors declare that they have no competing interests.

## Authors' contributions

RG conceived of the study, participated in design, analysis, and interpretation of data, and drafted the article. EC participated in analysis and interpretation of data. SEL participated in statistical analysis and interpretation of data. DR participated in conception and design. GT participated in conception and design. CH participated in conception, design, analysis, and interpretation of data. All authors revised the article critically, and read and approved the final manuscript.

## Pre-publication history

The pre-publication history for this paper can be accessed here:

http://www.biomedcentral.com/1471-2458/11/796/prepub

## Supplementary Material

Additional file 1 **Story themes and elements in previous analyses and present study**. the coding frames of Wahl *et al *and Corrigan *et al*'s studies alongside the codes that were included in this study. Click here for file

Additional file 2 **Longitudinal study codebook**. codebook used for the content analysis in the study, describing in detail the criteria for all codes. Click here for file
