# Supplementary material for: Newspaper coverage of mental illness in the UK, 1992-2008
Source: BMC Public Health. 2011 Oct 12;11:796. doi: 10.1186/1471-2458-11-796 (PMC3205064; doi:10.1186/1471-2458-11-796)
Supplement: Additional file 1 — Story themes and elements in previous analyses and present study. the coding frames of Wahl et al and Corrigan et al's studies alongside the codes that were included in this study. [file 1471-2458-11-796-S1.PDF]

STORY THEMES AND ELEMENTS IN PREVIOUS ANALYSES AND PRESENT STUDY

**Wahl et al. (2002)**	**Corrigan et al. (2005)**	**Present study**
Themes	*Themes*	*Themes*
Dangerousness	Blame	Bad news
Unfair treatment	Personal blame	Danger to others
Treatment is successful	Parental failure	Suicide and self-injury
Personal success occur	Genetic or biological cause	Victimization and severe mistreatment
Biogenetic causes	Environmental cause	Strange, inept, or burdensome
Advances in research	Dangerousness	Good news
Qualified dangerousness	Danger to others	Understanding mental illness
Parental causation	Violent crime	Explaining: causes, treatments, prevalence, and symptoms
Need for more resources	Nonviolent crime	Biological
Stigma/public education	Suicidal or self-injurious behavior	Psychosocial
Parity/insurance	Mental illness as legal defense	Not specified
Homelessness	Legal competence	Individuals and groups affected by mental illness
Legal issues	Criminal victimization	Services and advocacy
	Drug and alcohol abuse	Mental health service inadequacies and improvements
Elements	Treatment and recovery	Stigma, discrimination, and public education
Consumer biography	Research advances	
Generic references to mental illness	Biological treatments	*Elements*
Opinions of advocacy representatives	Psychosocial treatments	Notes that effective treatment is available and recovery is possible
Opinions of medical experts	Recovery as an outcome	Notes that mental illness is relatively common
Family perspectives	Advocacy actions and concerns	Features a direct quote from an individual with mental illness
Reference to people as their disorders	Poor-quality treatment	Features a pejorative slang term
Use of People First Language	Shortage of resources	
Consumer perspectives	Homelessness	
Referrals for information/help	Housing issues	
Use of slang expression	Insurance parity	
Opinions of non-medical experts		
Misuse of psychiatric terms		
