# Supplementary material for: Newspaper coverage of mental illness in the UK, 1992-2008
Source: BMC Public Health. 2011 Oct 12;11:796. doi: 10.1186/1471-2458-11-796 (PMC3205064; doi:10.1186/1471-2458-11-796)
Supplement: Additional file 2 — Longitudinal study codebook. codebook used for the content analysis in the study, describing in detail the criteria for all codes. [file 1471-2458-11-796-S2.PDF]

LONGITUDINAL STUDY CODEBOOK

Abbreviations: MI = mental illness; PWMI = people with a mental illness

DIAGNOSIS

What mental illnesses are discussed in the article?

- In general, code *all* diagnoses mentioned in the article. Most articles only likely to contain one.
- However, if an article is focused throughout on one particular diagnosis, and then a second diagnosis is mentioned once in passing, only code the main diagnosis.
- If there are more than one of the same type of disorder, only code once e.g. if both anorexia and bulimia are mentioned, only code 'eating disorders' (4) once.
- The diagnosis has to be named or very unambiguously referred to.
- How to code...
    - Seasonal affective disorder: code as depression (1).
    - Phobias: code as anxiety disorders (7).
    - 'Psychopathic disorder': code as personality disorder (11).
    - 'Melancholy': code as depression (1).
    - Panic attacks: if they're described as an illness or they are chronic, code as anxiety disorders (7).
    - Do not code: neurodegenerative and neurodevelopmental disorders, ME/CFS, and addiction. Any other disorder not listed (e.g. 'dissociative disorder', 'conduct disorder') code as other (15).
- Sometimes an individual will be described as having a disorder with a general term at the start (e.g. 'depression'), but later in the article a more specific diagnosis is given (e.g. 'post-natal depression' or 'bipolar'), in which case just code the more specific diagnosis.

<u>THEMES</u>

What is the story about?

- Code the single, most prominent theme in the article.
- Articles are often somewhat ambiguous: they could arguably be coded as various themes. In these cases of ambiguity – is it this theme or that theme? – try and base it on what's mentioned in the headline. If that doesn't help – i.e. the headline mentions no theme or more than one theme – which theme is discussed at greater length in the article? If they're both discussed at equal length, which comes first in the piece?

BAD NEWS

- The articles that most unambiguously come here are stores about 'one-off' negative incidents involving someone with a mental illness e.g. a murder, a suicide. However, there are other articles coded here which aren't linked to a specific incident.

11 Danger

- Any involvement in a violent or threatening crime – murder, assault, terrorism, stalking etc. – by PWMI, or which more generally talks about mental health of those perceived as dangerous.
- Code here even if the onset of the illness *follows* the crime, as it still 'makes the link' and it could be deduced that they were always prone/at risk.
- Court cases where the individual's illness is not certain:
    - As long as there is a reasonable suspicion or it is discussed at length, code here.
    - Articles about someone pretending to have a mental illness to 'get off' are also coded here.
    - Articles about individuals who have falsely confessed to a crime because of an illness, however, are coded 14 (see below).
- Prisoners' mental health and special hospitals
    - Articles about prisoners' mental health might be sympathetic and say they deserve better treatment. However, it is still coded here *unless* it goes so far to say that PWMI should not be in prison at all, but rather should be patients in a hospital.
    - Articles on special hospitals generally come here, especially if they describe patients in criminal terms – 'offenders', 'prisoners', 'dangerous' etc. However, the article might be about acts of abuse or mistreatment by staff (13) or suicide (12), in which case code as that. The question is: *why* is this being reported? In general, danger is a driver for reporting on special hospitals – even when it's ostensibly sympathetic. However, if patients are described correctly as 'patients' (or services users), and it's about the poor quality of services (31), then code accordingly.

- As with all themes, this can be mentioned in an article but not be the primary focus, hence is not coded.

12 Suicide and self-injury

- If it's about suicidal or self-injurious acts, it comes here.
- Refusing treatment to a life-threatening degree (e.g. with anorexia) is coded here.
- Somebody being bullied or pushed into suicide is coded here.
- Stories in which it is strongly suggested but not explicitly said – e.g. a man who fell to his death and had depression – are coded here.
- However, if suicide is used as a measure of population-wide mental health, and the illnesses leading to suicide and their treatment/prevention are discussed more than the prevalence and acts themselves, it could come under another theme e.g. explaining (21-23), services (31), or public education (32).
- Suicidal 'thoughts' are not sufficient reason to code here; this is more often grounds for just talking about depression. There has to be an attempted or completed suicide, or a very clear intent which is only stopped by someone else. However, even a story about an individual who attempts/completes suicide may be coded as 'Individuals affected by MI' (24), provided the suicide is not mentioned in the headline, and other aspects of their life are discussed at greater length than the suicidal act.
- 'Suicide by cop' stories can be danger (11), suicide (12), or – if it suggests they were wrongly shot – mistreatment (13). As always, judge what the emphasis is based on what the headline says.

13 Criminal victimization and severe mistreatment

- Can occur within care (e.g. abuse of patients, serious neglect) or outside.
- However, if the tone is more positive and the mistreatment is not severe e.g. somebody campaigning against poor treatment (as opposed to abusive treatment) within care, it might be better coded as 'services' (31).
- Incidents in which there is no perpetrator i.e. accidents, or the mistreatment is less severe i.e. misfortune, likely comes under 'Strange, inept...' (14).

14 Strange, inept, or burdensome

- This is a good 'catch all' category for a story which seems negative but doesn't neatly fit one of the other 3 bad news categories. Generally it's about portrayals of PWMI as strange, pathetic, incapable, irritating i.e. less severe forms of problematic behaviour than the other bad news categories.
- Common story types
    - Falsely claiming an illness for personal gain or attentions comes here e.g. a story about a compensation payout for workplace stress when the tone of the article is sceptical. However, if the tone is sympathetic, code as 'Individual affected...' (24). Also, if someone was actually dismissed because of their illness, it could be 'Stigma, discrimination...' (32).

    - Stories which imply personal responsibility for an MI.
        - Stories about drugs being a cause of mental illness are the most common e.g. cannabis and schizophrenia.
    - False confessions to crime by PWMI.
    - Non-violent crime.
    - Articles which mock mental illness.
    - Descriptions of fictional portrayals (e.g. a film review) which portray PWMI as bizarre/comical, if the reviewer uncritically reports it. However, if the review is critiquing this stigmatizing view, it can come under 'Stigma, discrimination…' (32).
    - Stories about PWMI going missing.
    - Stories about people being sectioned when the reason is unclear. When the reason is clearly because of danger towards self or others, however, code as danger (11; look out for terms like 'risk', 'disturbed' etc.) or suicide (12).
    - Stories about milder victimization or misfortune than those come under 13.
- Articles which look like they're 'Explaining' (21-23) or 'Individual affected by...' (24) might in fact come here, if...
    - It's about a spiral into failure, 'fall form grace' etc.
    - The focus is on the disruption someone with an MI causes. However, if this is in the context of advice and addressing the problem and sensitively explaining the behaviour, then code as Explaining.
    - They suggest the individual really brought it upon themselves (though these are rare).
    - Essentially the distinction is: is this sympathetic or not? Look out for negative terms like 'tragic', 'tormented', 'tortured' etc.

UNDERSTANDING MENTAL ILLNESS

Explaining

- Articles about causes, treatments, prevalence, symptoms of MI.
- Common article types: news articles about research, health features, medical advice columns, non-fiction book reviews.
- Does it talk about the biological (21) or psychosocial (22) aspects of MI?
    - If it talks about *both* biological and psychosocial factors, judge which is spoken about in greatest detail, comes in the headline, or is given more support to by the journalist and sources. If you really can't pick i.e. it is emphasized that *both* matter and *both* are discussed at equal length, just code whatever comes first.
    - If it talks about a biological or psychosocial aspect only to reject it e.g. an article about SSRIs saying that they don't work, code it as the opposite or 'not-specified' e.g. if it says SSRIs don't work but CBT *does*, code 'Psychosocial'; if it says SSRIs don't work but doesn't mention an alternative, code 'Not specified' (23).

  - The discussion of the biological/psychosocial treatment needn't be extensive to be coded as one or the other. An article which talks mostly about the symptoms of an illness, even if in just one sentence it mentions a particular cause or treatment, code base on that mention.
- Articles about the treatment of mental illness can be ambiguous in terms of whether they come here, or in services (31). The question is, is it *primarily* about the *therapies* – do they work, how they work – or is it about the *services* – funding, availability, organisational aspects (e.g. community care vs. institutions), staff training etc.

21 Biological

- Articles discussing MI from the perspective of genetics, physiology (esp. neuroscience), or pharmaceutical treatments.
- Alternative *medicine* (e.g. St John's wart) comes here. Alternative *therapies* like exercise, aromatherapy and light boxes come here if they talk about it in physiological terms (e.g. 'exercise increases serotonin'), otherwise 'Psychosocial' (as it's generally implied that they work on your 'mind'/feelings). Homeopathy depends also on how it's discussed, whether it's described as a medical treatment or just as placebo, hence psychosocial.
- Chemical causes of an illness (e.g. depression as a side-effect of a medication for heart problems, or pollution etc.) come here.
- Talking about the biomedical consequences of a MI (e.g. increased risk of heart disease among those with depression) *don't* come here: these are Explaining: Not specified (unless it also discusses a biological cause/treatment of the MI).

22 Psychosocial

- Articles discussing MI from the perspective of psychosocial causes and psychotherapeutic treatments.
- Causes like stress, social pressures, childhood trauma, and treatments like psychotherapy or lifestyle changes.
- Articles about the ineffectiveness or inappropriateness of biomedical treatments come here provided it suggests that the cause/solution is more psychosocial (otherwise it might be not-specified).
- For stories about PTSD and post-natal depression, the immediate trigger (i.e. trauma or birth) does not count as a 'psychosocial' cause. Need something more e.g. saying PND caused by lack of emotional support during pregnancy (psychosocial) or hormonal changes (biological [21]). However, stories about these disorders are often about specific individuals or groups who have suffered a specific trauma, not about the illnesses in general, hence code as 24.
- Placebo effect comes here.

23 Not-specified

- Articles which talk about illnesses without *any* reference to their causes or treatment.

- Most likely to occur in the context of articles discussing an MI's symptoms or consequences. Articles discussing the prevalence of an illness might also come here, though they often mention a psychosocial cause.
- When the 'cause' is simply a history of another mental illness – e.g. having depression in the past increases risk of PTSD after trauma – it's coded here (unless there's an explanation of that original illness).

24 Individuals and groups affected by mental illness

- When it's about individuals or groups and doesn't seek to make wider claims about mental illness.
- 'Groups' refer to professional groups – e.g. soldiers, doctors – or self-selected population sub-groups e.g. fans of a particular hobby. General population groups such as women, children, teenagers, schoolchildren, older men etc. (i.e. age and gender groups), students, are so broad that articles about them come under explaining (21-23).
- However, sometimes individuals can be used as examples to 'explain' the illness in general; its causes, treatments etc.: hence code 21-23, e.g. if an article opens with a general claim 'rising numbers of young people are suffering depression, this is one person's story'. How to decide whether it's just about an individual, or whether it's making more general claims (explaining):
    - What's in the headline/opening paragraph? If there's *any* indication of it being general (e.g. 'one of a rising number of…', 'the effects of Prozac explained by one person who has taken it...') then it's explaining.
    - Problem pages: medical problem pages are explaining (check if the byline is a Dr), but non-medical are usually individual.
    - Stories about celebs more likely to be individual, while stories about individual members of the public more likely to be used as indicative of the illness in general. This is just a rough guideline.
    - Essentially: is it *about* the individual or the illness?
- Equally, if there's a story about a professional group being affected by a cause which affects us all e.g. 'the effects of the recession on city workers', it comes under explaining (21-23). This tends to be less common than the individuals as examples.
- If the focus is on the poor services received by an individual or a group, it is coded as services (31); equally if it's about discrimination or stigma faced by an individual or a group, it's coded 32.
- Descriptions of fictional portrayals (e.g. a film or book review) will often come under this, provided they're non-sensational and broadly sympathetic. Otherwise it might be 'Strange' (14) or 'Danger' (11) etc.
- Stories about PTSD, whether about individuals, a group of survivors of a particular incident, or a professional group (e.g. soldiers), often come here.
- Generally the account has to be mainly sympathetic and non-sensationalist to be coded here. If it's better accounted for by one of the bad news categories, then code accordingly. As always, the question is: what is the primary focus?

- This includes articles which aren't really *about* mental illness, more about 'an individual who happens to have an MI' e.g. a review of an album by a musician in which it's mentioned they have an illness.
- Obituaries of PWMI might come here. However, if they were particularly famous as campaigners, it might come under services (31) or stigma (32). Obituaries of psychiatrists, meanwhile, likely to be 'explaining' – if it discusses contributions they made to the medical side of treating MI – or 'services' – if it discusses contributions they made to the organisational side of treating MI.

SERVICES AND ADVOCACY

31 Health service inadequacies and improvements

- About mental health policy and the resource (funding, availability) and organisational aspects (e.g. community care vs. institutions, staff training) of MI.
- This can be about services in general or services as they apply to a particular group or individual.
- An article about a new hospital opening, a new service being launched – e.g. a website with information for PWMI – whether by government or mental health charities. When the new 'service' is in fact a new kind of therapy (psycho- or pharmaco-), see if the article is more about the therapy's effectiveness or its increased availability (judge based on the headline, whether or not most quotes come from politicians or doctors etc.).
- A more general complaint about the nature of psychiatric treatment would also come here e.g. psychiatrists have too much power over services users.
- This includes wider services such as housing as they apply to PWMI.
- Stories about people taking time off work can come here if they're sympathetic and about the inadequate support offered from employers. Otherwise the might be 'Strange, inept...' (14) if unsympathetic, or 'Stigma, discrimination...' (32) if it's about someone losing their job because of their MI.
- Stories about patient empowerment / service user involvement in services come here.

32 Stigma, discrimination, and public education

- Any discussion of stigma and discrimination. Remember, however, that anything to do with inadequate care – inc. discrimination within the health system – comes under services (31). This is more for employment discrimination, prejudicial attitudes etc.
- Campaigning efforts to reduce ignorance and increase understanding of mental illness. This will often involve a lot of explaining about MI, which might be expected to go under explaining (21-23). However, this category is for instances where there is an active campaign to increase understanding – such as from a charity or the RCPsych – as opposed to research which is finding out more about MI.
- Articles about the ridicule/mocking PWMI face in which that ridicule is clearly condemned (e.g. a film review which criticizes a negative portrayal).

ELEMENTS

Anything from none to all of these can be coded for any story. The reference can be as brief or as long as possible.

EFFECTIVE TREATMENT AND RECOVERY

- Mentions that a treatment is effective or notes that someone has recovered or seen a significant improvement in their symptoms.
- There has to be unambiguous support for the effectiveness: 'It is claimed to improve mental health...' wouldn't be quite enough. It doesn't have to quote research however: it simply has to be a statement by the journalist or source saying that something works (unless the journalist overtly refutes what the source has said) or someone has recovered.
- Includes someone talking about their illness as clearly something in the past which is no longer around, even if they don't describe the actual process of recovery.
- Has to be an effective *treatment*, not the *preventative* effects of something.
- Being 'up/recovered' from a temporary bout of illness, but in which relapse is suggested as being likely, is not enough.

SERVICES USER VOICE

- A direct quote >20 words from an individual with mental illness (doesn't have to be contiguous) *about* mental illness or something directly related. When the individual is not talking about their illness but something unrelated e.g. about a crime they committed, this is not coded.
- Include people from service user organisations like the Hearing Voices Network, Manic Depressive Fellowship, or the Depression Alliance (but not mental health charities like Mind or Rethink).
- A 'quote' from a fictional character does not count.

MENTAL ILLNESS IS COMMON

- This can be a specific number (e.g. 1 in 4) or a more general comment like 'mental illness is common'.
- If numbers are specified, it has to be more than 1 million or 1 in 20 / 5% (of the general population or of a gender/age group).
- If a word is used, it has to be absolute e.g. 'large numbers', 'common', and not relative e.g. 'rising numbers'.
- This doesn't have to be about the whole population. It can be the proportion of any gender or age group e.g. 1 in 10 older women, over 1 million teenagers etc. It's the same as the distinction in terms of whether a story is about 'Groups' (24) or whether it's 'Explaining' (21-23) i.e. rates of illness among professional groups wouldn't be coded.
- Has to be about the number of people with the actual diagnosis, not just the number with certain related attitudes or symptoms – e.g. saying '1 in 4 teenage

girls are worried about their weight' in an article about anorexia is not coded. Equally proportions of people committing self-harm wouldn't be coded. However, saying that e.g. '1 in 5 take antidepressants' would be coded, as that unambiguously relates to having a clinical disorder.

PEJORATIVE LANGUAGE

- Any of the following terms:
  - Crazy/crazed
  - Mad (saying someone is mad, not just using the term 'madness')
  - Deranged
  - Lunatic/Loony
  - Psycho/psychopath (unless it says 'psychopathic personality disorder')
  - Nutter
  - Berserk
  - Maniac
  - Bonkers
- NOT 'insane' because it still a legitimate legal term.
